# Update: Interim Guidance for Preconception Counseling and Prevention of Sexual Transmission of Zika Virus for Men with Possible Zika Virus Exposure — United States, August 2018

**DOI:** 10.15585/mmwr.mm6731e2

**Published:** 2018-08-10

**Authors:** Kara D. Polen, Suzanne M. Gilboa, Susan Hills, Titilope Oduyebo, Katrin S. Kohl, John T. Brooks, Alys Adamski, Regina M. Simeone, Allison T. Walker, Dmitry M. Kissin, Lyle R. Petersen, Margaret A. Honein, Dana Meaney-Delman

**Affiliations:** ^1^Division of Congenital and Developmental Disorders, National Center on Birth Defects and Developmental Disabilities, CDC; ^2^Division of Vector-Borne Diseases, National Center for Emerging and Zoonotic Infectious Diseases, CDC; ^3^Division of Reproductive Health, National Center for Chronic Disease Prevention and Health Promotion, CDC; ^4^Division of Global Migration and Quarantine, National Center for Emerging and Zoonotic Infectious Diseases, CDC; ^5^Division of HIV/AIDS Prevention, National Center for HIV/AIDS, Viral Hepatitis, STD, and TB Prevention, CDC.

## Abstract

Zika virus infection can occur as a result of mosquitoborne or sexual transmission of the virus. Infection during pregnancy is a cause of fetal brain abnormalities and other serious birth defects (*1*,*2*). CDC has updated the interim guidance for men with possible Zika virus exposure who 1) are planning to conceive with their partner, or 2) want to prevent sexual transmission of Zika virus at any time (*3*). CDC now recommends that men with possible Zika virus exposure who are planning to conceive with their partner wait for at least 3 months after symptom onset (if symptomatic) or their last possible Zika virus exposure (if asymptomatic) before engaging in unprotected sex. CDC now also recommends that for couples who are not trying to conceive, men can consider using condoms or abstaining from sex for at least 3 months after symptom onset (if symptomatic) or their last possible Zika virus exposure (if asymptomatic) to minimize their risk for sexual transmission of Zika virus. All other guidance for Zika virus remains unchanged. The definition of possible Zika virus exposure remains unchanged and includes travel to or residence in an area with risk for Zika virus transmission (https://wwwnc.cdc.gov/travel/page/world-map-areas-with-zika) or sex without a condom with a partner who traveled to or lives in an area with risk for Zika virus transmission. CDC will continue to update recommendations as new information becomes available.

## Review of Evidence

Primarily transmitted through the bite of an infected *Aedes aegypti* mosquito, Zika virus can also be transmitted through unprotected sex (i.e., without correct and consistent use of a condom) with an infected partner. As of July 3, 2018, 52 cases of confirmed sexual transmission of Zika virus infection have been reported in the United States since 2015 (https://www.cdc.gov/zika/reporting/case-counts.html). Most documented reports of sexual transmission have involved transmission from a man to a woman (*4*); however, transmission also has been reported from a man to another man (*5*) and from a woman to a man (*6*).

Despite their limited generalizability to humans, preliminary data from animal studies suggest that sexual transmission of Zika virus during pregnancy might pose a higher risk to the fetus than mosquitoborne transmission. In female rhesus macaques, vaginal inoculation (as a model for sexual transmission) of Zika virus appeared to enhance viral dissemination to the female reproductive tract, compared with subcutaneous inoculation (*7*). In an immunodeficient mouse model, poorer maternal outcomes and higher fetal viral titers were observed when exposure was through sexual transmission rather than subcutaneous or intravaginal infection (*8*). Prevention of sexual transmission of Zika virus during pregnancy can reduce the risk for maternal infection and the potential for congenital Zika syndrome.

The risk for congenital Zika syndrome associated with maternal Zika virus infection during the periconceptional period is not known. Maternal infection with other viruses (e.g., rubella) during the periconceptional period have been associated with infection in the fetus and adverse pregnancy outcomes; although in some cases, the timing of infection relative to conception was uncertain (*9*–*13*). To date, there are no published data definitively linking Zika virus infection around the time of conception to adverse pregnancy outcomes.

Since the last update of this guidance on October 7, 2016 (*3*), additional evidence relevant to the assessment of risk for sexual transmission of Zika virus infection has been reported. A literature search of PubMed was performed to identify new human studies and data published in English since October 2016. References for included articles were also screened. Specific search terms used included “sexual transmission” or “semen” or “seminal fluid” and “Zika.” The search yielded 15 publications, including case reports, case series, and nine cohort studies, which were reviewed for new, primary data.

Among the currently available reports of sexual transmission of Zika virus, the longest period from symptom onset in the index case to potential sexual transmission to a partner was between 32–41 days (*14*); most reports indicate much shorter intervals (*4*). The longest period after symptom onset at which replication-competent (i.e., potentially infectious) virus has been detected in semen by culture or cytopathic effect was 69 days (*15*). No other studies reported potentially infectious Zika virus in semen specimens obtained ≥40 days after symptom onset (*16*–*33*).

Numerous publications have reported on the detection of Zika virus RNA in semen (*4*,*15*–*41*), although this might not indicate the presence of infectious virus at the time of sampling or correlate with the potential for sexual transmission of infectious virus. In the largest published cohort study to date, involving 184 men with confirmed symptomatic Zika virus infection from whom a baseline specimen and serial semen specimens were collected at 2-week intervals, Zika virus RNA shedding in semen declined during the 3 months after symptom onset (*28*). Overall, Zika virus RNA was detected in semen in 61% (22 of 36); 43% (48 of 112); and 21% (28 of 131) of participants from whom specimens were collected within 30, 31–60, and 61–90 days of illness onset, respectively. At >90 days after illness onset, semen of ≤7% of participants had detectable Zika virus RNA. The estimated mean time to clearance of Zika virus RNA from semen was 54 days (*28*). Another large cohort study conducted in Puerto Rico followed 117 men, 89 of whom provided semen specimens and reported similar results: at >90 days after illness onset 11% (8 of 74) of men had detectable RNA in semen (Gabriela Paz-Bailey, Division of Vectorborne Diseases, National Center for Emerging and Zoonotic Infectious Diseases, CDC; personal communication, 2018) (*32*). Similar findings have been observed in smaller cohort studies (*16*,*17*,*23*,*25*,*29**,**37**,**39*). Zika virus RNA has been detected in semen for as long as 370 days after symptom onset (*17*); however, detection for long periods is rare. Limited data suggest the incidence of Zika virus RNA shedding in semen and its persistence after infection are likely similar for symptomatic and asymptomatic men infected with Zika virus (*40*–*42*).

## Guidance for Preconception Counseling and Prevention of Sexual Transmission

CDC’s last interim guidance released in October 2016 was based on the maximum duration of detection of Zika virus RNA in semen. In the last interim guidance, CDC recommended that men with possible Zika virus exposure wait at least 6 months after symptom onset (if symptomatic) or their last possible Zika virus exposure (if asymptomatic) before trying to conceive with their partner (*3*). New data published since then support an update to that interim guidance. CDC now recommends that men with possible Zika virus exposure who are planning to conceive with their partner wait for at least 3 months after symptom onset (if symptomatic) or their last possible Zika virus exposure (if asymptomatic) before engaging in unprotected sex. CDC now also recommends that for couples who are not trying to conceive, men can consider using condoms or abstaining from sex for at least 3 months after symptom onset (if symptomatic) or their last possible Zika virus exposure (if asymptomatic) to minimize their risk for sexual transmission of Zika virus. Recommendations for men with possible Zika virus exposure whose partner is pregnant remain unchanged; these couples should be advised to consistently and correctly use condoms during sex or abstain from sex for the duration of the pregnancy (Table).

**TABLE:** CDC recommendations for preconception counseling and prevention of sexual transmission of Zika virus among persons with possible Zika virus exposure — United States, August 2018

Exposure scenario	Recommendations (update status)
Only the male partner travels to an area with risk for Zika virus transmission and couple planning to conceive	The couple should use condoms or abstain from sex for at least 3 months after the male partner’s symptom onset (if symptomatic) or last possible Zika virus exposure (if asymptomatic). **(Updated recommendation)**
Only the female partner travels to an area with risk for Zika virus transmission and couple planning to conceive	The couple should use condoms or abstain from sex for at least 2 months after the female partner’s symptom onset (if symptomatic) or last possible Zika virus exposure (if asymptomatic). (No change in recommendation)*
Both partners travel to an area with risk for Zika virus transmission and couple planning to conceive	The couple should use condoms or abstain from sex for at least 3 months from the male partner’s symptom onset (if symptomatic) or last possible Zika virus exposure (if asymptomatic). **(Updated recommendation)**
One or both partners have ongoing exposure (i.e., live in or frequently travel to an area with risk for Zika virus transmission) and couple planning to conceive	The couple should talk with their health care provider about their plans for pregnancy, their risk for Zika virus infection, the possible health effects of Zika virus infection on a baby, and ways to protect themselves from Zika. If either partner develops symptoms of Zika virus infection or tests positive for Zika virus infection, the couple should follow the suggested timeframes listed above before trying to conceive. (No change in recommendation)*
Men with possible Zika virus exposure whose partner is pregnant	The couple should use condoms or abstain from sex for the duration of the pregnancy. (No change in recommendation)*

* Petersen EE, Meaney-Delman D, Neblett-Fanfair R, et al. Update: interim guidance for preconception counseling and prevention of sexual transmission of Zika virus for persons with possible Zika virus exposure—United States, September 2016. MMWR Morb Mortal Wkly Rep 2016;65:1077–81.

CDC continues to recommend shared patient-provider decision making, in which couples and health care providers work together to make decisions about timeframes to wait before trying to conceive after possible Zika virus exposure. Some couples might choose to wait shorter or longer periods after possible Zika virus exposure, based on individual circumstances (e.g., age, fertility, or details of possible exposure), clinical judgment, and a balanced assessment of risks and expected outcomes. Other guidance for preconception counseling and prevention of sexual transmission of Zika virus after possible Zika virus exposure remains unchanged (*3*).

**Summary**

**What is already known about this topic?**

Zika virus infection during pregnancy is a cause of serious birth defects. CDC previously released interim guidance on preconception counseling and prevention of sexual transmission of Zika virus in October 2016.

**What is added by this report?**

CDC now recommends that men with possible Zika virus exposure who are planning to conceive with their partner wait at least 3 months after symptom onset or their last possible Zika virus exposure before engaging in unprotected sex. This updated timeframe also applies to prevent sexual transmission of Zika virus.

**What are the implications for public health practice?**

These recommendations provide couples planning pregnancy with updated timeframes expected to reduce the risk for fetal Zika virus infection.
